# The role of combining medroxyprogesterone 17-acetate with human menopausal gonadotropin in mouse ovarian follicular development

**DOI:** 10.1038/s41598-018-22797-6

**Published:** 2018-03-13

**Authors:** Xiaowei Wen, Jun Xie, Lixia Zhou, Yong Fan, Baofeng Yu, Qiuju Chen, Yonglun Fu, Zheng Yan, Haiyan Guo, Qifeng Lyu, Yanping Kuang, Weiran Chai

**Affiliations:** 1 Department of Assisted Reproduction, Shanghai Ninth People’s Hospital, Shanghai Jiaotong University School of Medicine, 639 Zhizaoju road, Shanghai, 200000 China; 2 Department of Biochemistry and Molecular Biology, School of Basic Medical Sciences, Shanxi Midical University, 56 Xinjian South Road, Taiyuan, Shanxi Province 030002 China

## Abstract

Medroxyprogesterone 17-acetate (MPA) combined with human menopausal gonadotropin (hMG) has been effectively used for ovarian stimulation in clinical practice. However, the molecular mechanism of MPA + hMG treatment in follicular development is poorly described. Here we performed a study to investigate the impact of MPA + hMG on ovarian stimulation utilizing a mouse model *in vivo*. Forty female BALB/C mice were randomly divided into four groups of 10 each and treated during ciestrus stage and continued for 5 days: control group, MPA group, hMG group, and MPA + hMG group. Morphological and molecular biology methods were used for detecting serum hormones and ovarian function. MPA + hMG group exhibited increasing follicle stimulating hormone (FSH), antral follicle, FSH receptor (FSHR) and phosphorylated mammal target of rapamycin (p-mTOR), and decreasing luteinizing hormone (LH), estradiol (E2), progesterone (P), corpus luteum, phosphoinositide 3-kinase (PI3K), Akt and mTOR compared with control group. In contrast, MPA + hMG group showed reduced FSH, LH, E2, P, corpus luteum, LH receptor (LHR), and activated PI3K,/Akt/mTOR pathway compared with hMG group (P < 0.05). Collectively, these data definitively established that MPA plus hMG may modulate the hormone, hormone receptor and PI3K/Akt/mTOR signaling pathway to influence follicular development in the mouse ovary. Our study provides overwhelming support for MPA + hMG as an effective treatment for infertility in women.

## Introduction

Infertility is one of the most common diseases in the world. Currently, more and more patients have to seek help from assisted reproductive therapy (ART) to have their own child^1^. The administration of gonadotropin-releasing hormone (GnRH) analogues, including GnRH agonists and GnRH antagonists, is used for preventing premature LH surges in pituitary desensitization among infertile patients in conventional controlled ovarian hyperstimulation^2^. However, this therapy has proven to have some limits because of the increased incidence of ovarian hyperstimulation syndrome (OHSS) by GnRH agonists and the rate of premature LH surges (0.34–38.3%) via GnRH antagonists^3,4^. Owing to the “freeze-all” strategies, a new ovarian stimulation regimen, MPA combined with hMG treatment was proposed by Dr. Kuang to inhibit premature LH surges and reduce the incidence of OHSS during follicular phase^5^. Our previous clinical studies have demonstrated that the MPA + hMG treatment is successful used in patients with normal ovarian response, advanced maternal age, low ovarian response, or polycystic ovary syndrome (PCOS)^6^, however, the molecular mechanism is not understood.

P is widely used for menstrual disorders, hormone replacement therapy and luteal support for pregnancy^7^ Animal research found that P improves follicular viability by increasing the levels of vascular endothelial growth factor and granulosa cell proliferation in large follicles and promotes the maturation of fish oocytes by promoting germinal vesicle breakdown^8–10^. In clinic, MPA is used as an alternative to GnRH analog to suppress a premature LH surge and avoid a low response of the hypothalamic-pituitary-ovarian axis (HPOA) during the follicular phase^11^. Unlike dydrogesterone, MPA does not disturb the measurement of serum progesterone. And the the administration of P during follicular phase had no negative effect on oocyte retrieval rates of the hMG + MPA treatment cycles based on frozen embryo transfer^12^. This suggests that MPA + hMG treatment is crucial for ovarian stimulation.

In most physiologic contexts, the PI3K/Akt signaling pathway is an important regulatory factor for cell proliferation and the initiation of oocyte growth^13^. Ovarian follicular growth is dependent on the growth and proliferation of granulosa cells. Indeed, it was found that FSH (the main component of hMG) could promote the rapid activation of PI3K pathway in ovarian granulosa cells, and PI3K catalyzes the production of PIP3 in the plasma membrane, leading to membrane recruitment, phosphorylation, and activation of downstream branched chain kinase Akt^14^. Meanwhile, it is known that Akt causes mTOR activation through a variety of mechanisms^15^. Activation of mTOR synergistically stimulate the growth of follicles^16^.

Our previous data indicate that MPA + hMG treatment does not impair the outcome of IVF/ICSI for FET. Based on our own and others’ work, we developed a mouse model of MPA + hMG treatment to investigate the role of MPA + hMG in follicular development and hypothesized that MPA + hMG may promote follicular development by regulating ovarian hormones, hormone receptors and PI3K/Akt/mTOR signaling pathways.

## Results

### Ovary weight index

**Figure 1:**
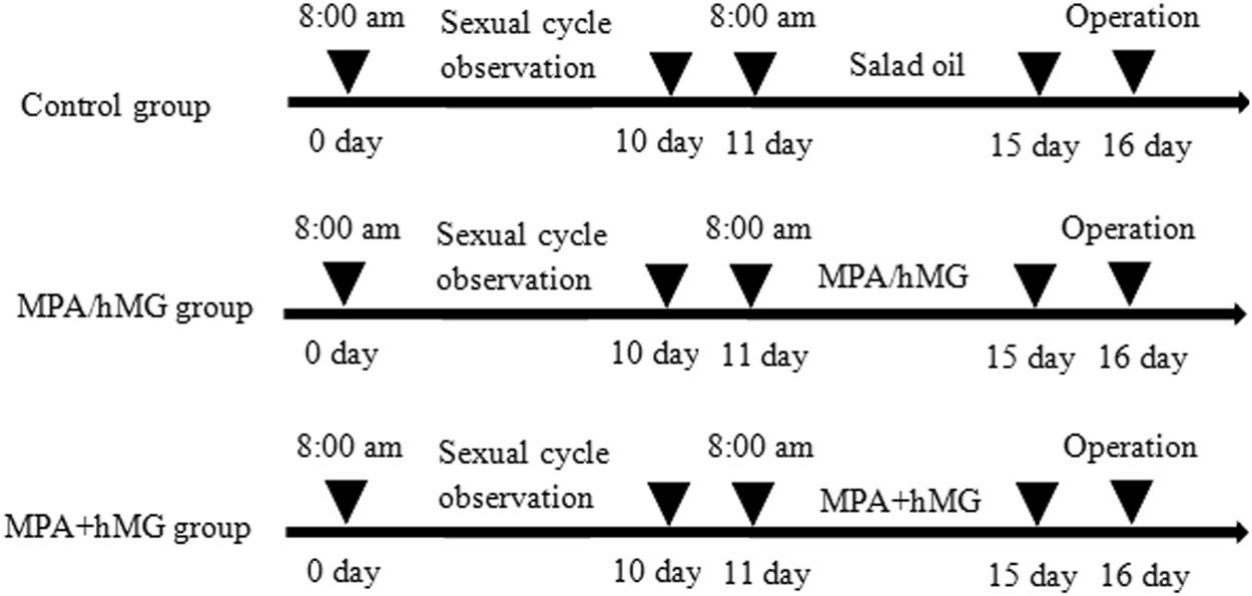
The establishment of MPA + hMG mice model.

After successful establishment of the MPA + hMG mice model, both ovaries of the mice were removed and weighed (Fig. 1). The ovarian weight index of each group is presented in Table 1. The ovarian wet weights and ovarian weight indexes are not significantly different between all groups (P > 0.05).

**Table 1:** The ovarian weight index of each group.

Group	Number	Ovarian wet weight (mg)	Ovary index (%)	P-value
Control group	10	9.72 ± 0.81	0.049 ± 0.041	0.2439
MPA group	10	10.22 ± 1.17	0.052 ± 0.058	0.3094
hMG group	10	10.74 ± 1.22	0.055 ± 0.061	0.2617
MPA + hMG group	10	11.66 ± 0.67	0.058 ± 0.033	—

Plus–minus values represent the mean ± SD. MPA, medroxyprogesterone acetate; hMG, human menopausal gonadotropin. Ovarian index (%) = ovarian wet weight (mg)/body weight (g) × 100%. P-value represents MPA + hMG group vs Control group, MPA group or hMG group.

### Serum hormone levels

**Figure 2:**
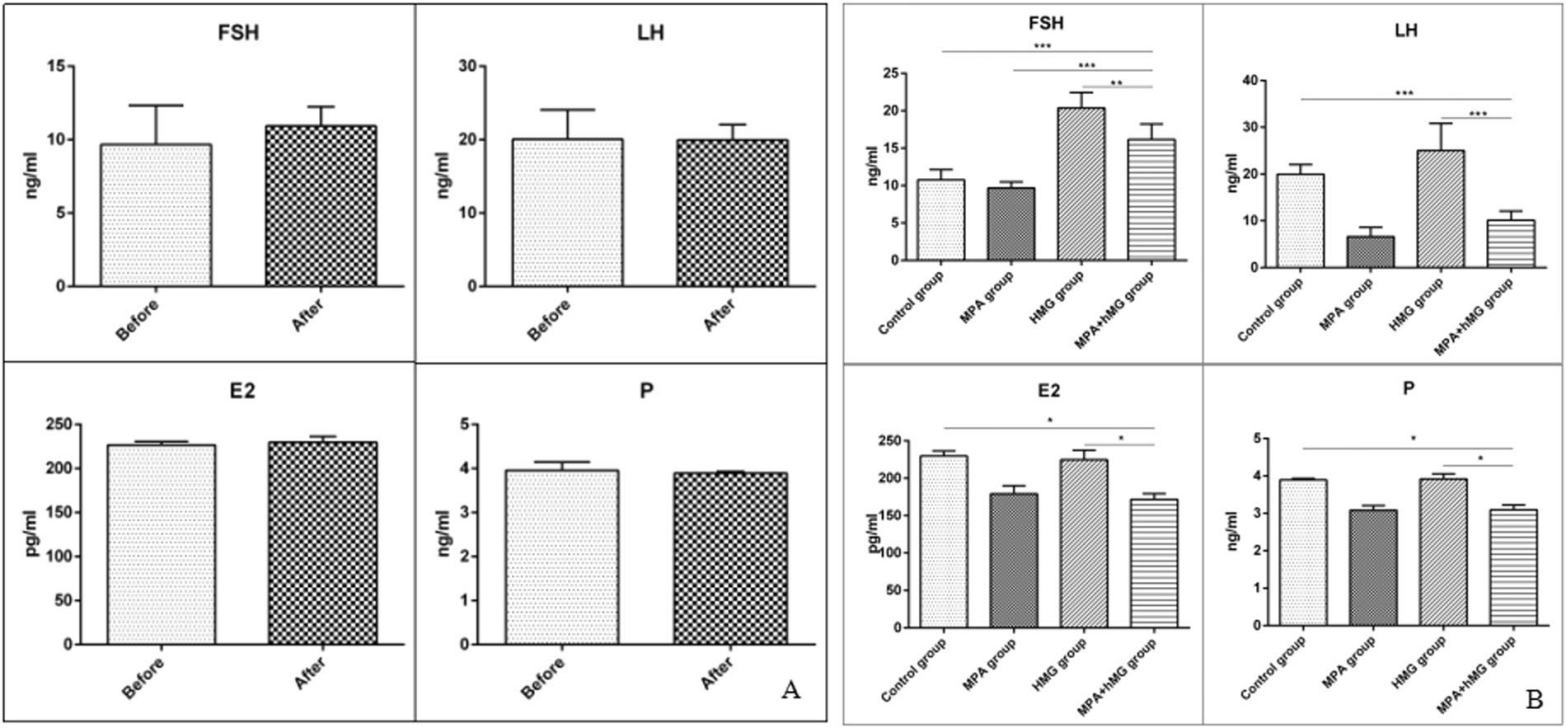
The changes in serum FSH, LH, E2 and P levels. (**A**) The level of serum FSH, LH, E2 and P in control group before and after 5 days; (**B**) The levels of serum FSH, LH, E2 and P were detected after 5 days. *Represents MPA + hMG group vs control group, MPA group or hMG group. *P < 0.05, **P < 0.01, ***P < 0.001.

To determine the effect of MPA + hMG on serum FSH, LH, E2, and P levels, serum was collected on the first day and after 5 days of treatment and these values were analyzed. In the control group, there are no significant changes in serum hormones before or after administration of salad oil (p-value is 0.3224, 0.9543, 0.3389 and 0.4665 in FSH, LH, E2, and P levels, respectively) (Fig. 2A). In the MPA + hMG group, there is a significantly increase in FSH level and decreases in LH, E2 and P levels compared to the control group (P < 0.05), meanwhile, FSH level is higher in MPA + hMG group than the MPA group. The levels of FSH, LH, E2, and P are all decreased in MPA + hMG group compared with the hMG group (P < 0.05) (Fig. 2B).

### Effects of MPA + hMG on ovarian follicle development

**Figure 3:**
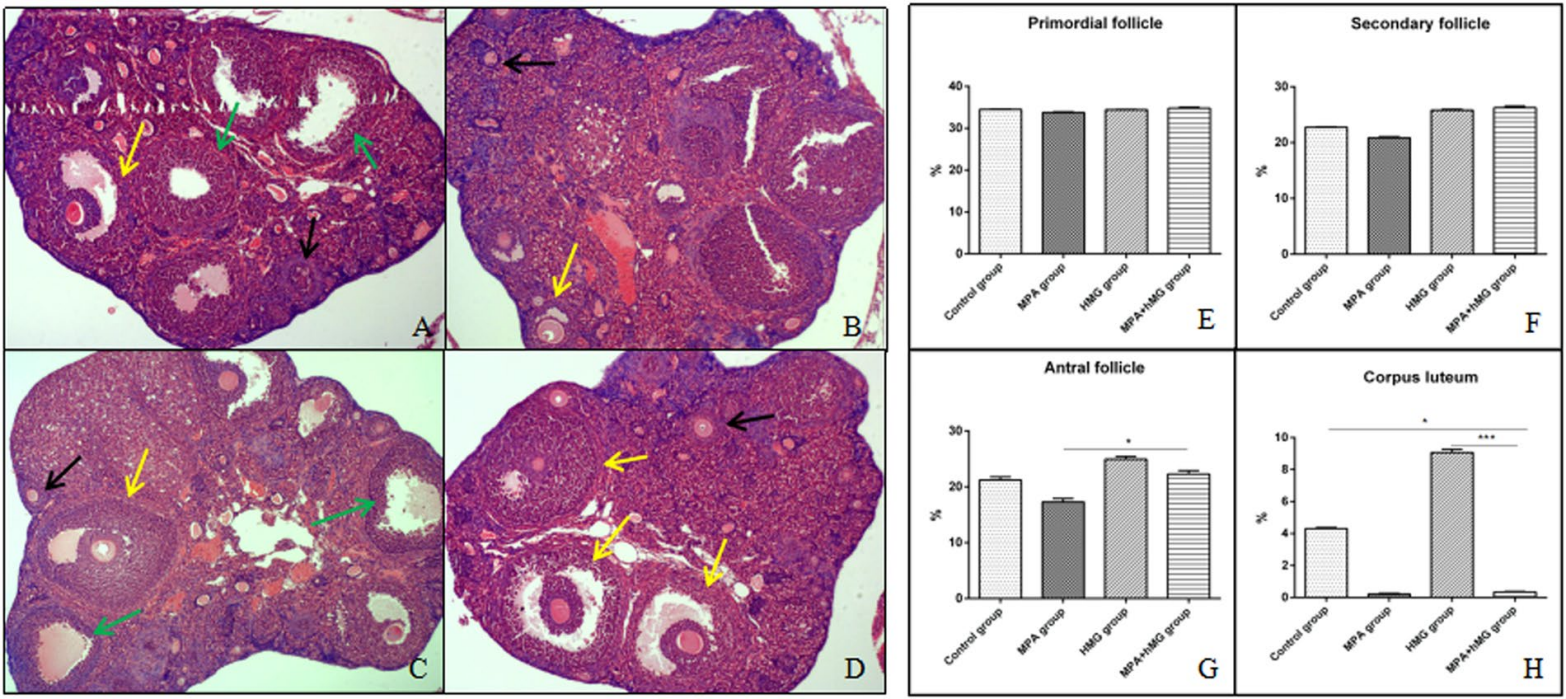
Effects of MPA + hMG on ovarian follicle development. Histologic sections were prepared from the ovaries of each group 24 hours after MPA or hMG treatment and stained with H&E, a detailed histopathological examination was performed under a light microscope noting each of the ovarian components. (**A**) Normal ovary of a control mouse. (**B**) The ovary with decreasing antral follicle and corpus luteum after MPA treatment. (**C**) The ovary with increasing corpus luteum after hMG treatment. (**D**) The ovary with increasing antral follicle and decreasing corpus luteum after MPA + hMG treatment. (**E**) The percentage of primordial follicle in four groups after 5 days of treatment. (**F**) The percentage of secondary follicle in four groups after 5 days of treatment. (**G**) The percentage of antral follicle in four groups after 5 days of treatment. (**H**) The percentage of corpus luteum in four groups after 5 days of treatment. Black arrows represent secondary follicle, yellow arrows represent antral follicle, green arrows represent corpus luteum. *Represents MPA + hMG group vs control group, MPA group or hMG group. *P < 0.05, ***P < 0.001.

In the control group (Fig. 3A), visible primordial follicles, greater volumes of the large follicles, thicker granulosa cells, and the limited amount of follicular atresia are observed; however, visible corpus luteum development is more pronounced than in the MPA + hMG group. In the MPA group (Fig. 3B), the number and volume of antral follicles are decreased compared to the control group; and there not appear newly formed corpus luteum in the MPA group. Greater volumes of the large follicles, thicker granulosa cells and corpus luteum are observed in the hMG group (Fig. 3C). In MPA + hMG group (Fig. 3D), the number of antral follicles and follicular volume is increased, granulosa cells are thicker, and the number of atresic follicles is decreased compared to the control group (P < 0.05); and there not appear newly formed corpus luteum compared with the control group and hMG group. There are not significantly different in the percentage of primordial follicles and secondary follicles between all groups (P > 0.05) (Fig. 3E,F). The percentage of antral follicles in MPA + hMG group is higher than that in MPA group (Fig. 3G). Meanwhile, corpus luteum is lower in MPA + hMG group compared to the control and hMG groups (Fig. 3H).

### The effects of treatment on ovarian FSHR, LHR, ER, and PR expression level

**Figure 4:**
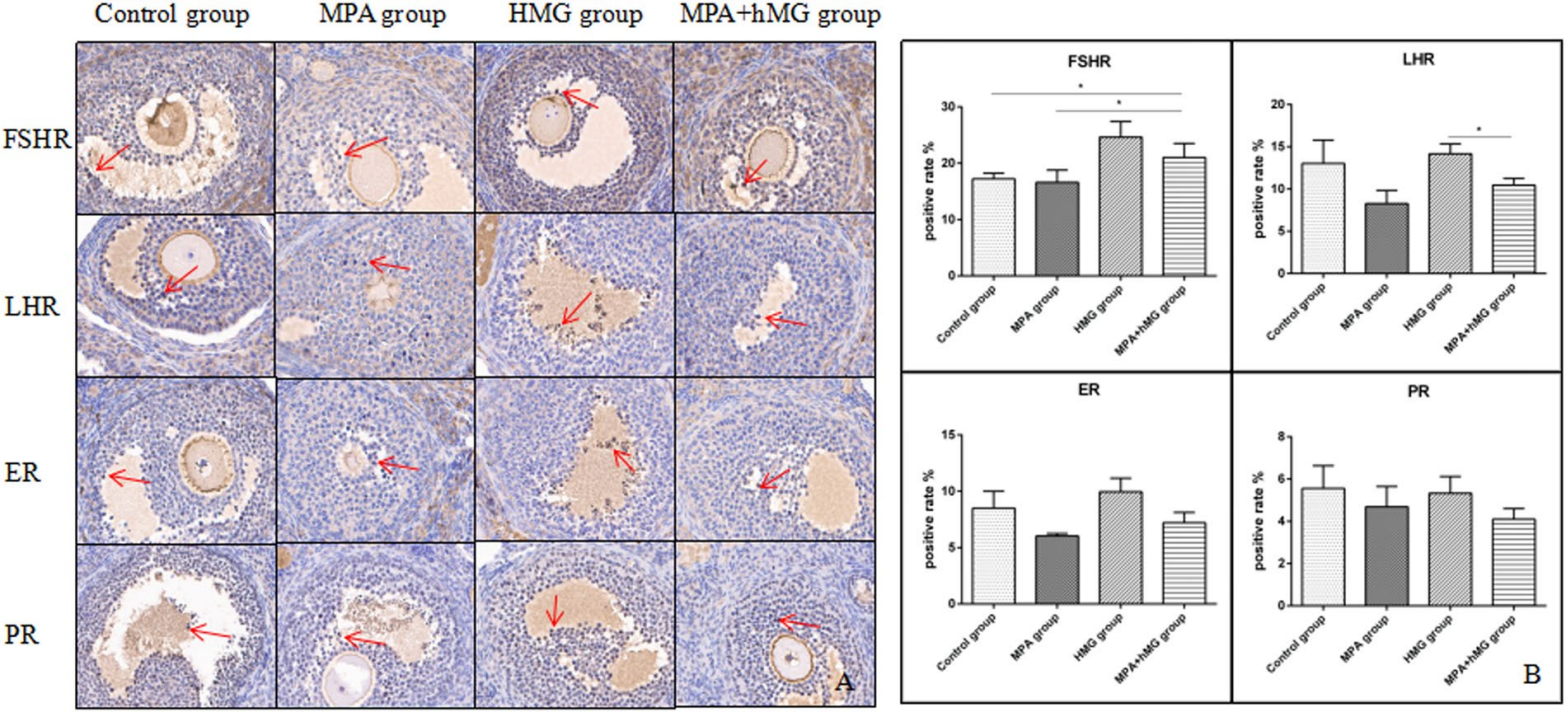
The effects of treatment on ovarian FSHR, LHR, ER, and PR expression level. (**A**) The FSHR, LHR, ER, and PR were detected by immunohistochemistry, red arrows represent the expression of receptors. (**B**) The positive rate of FSHR, LHR, ER, and PR in four groups. *Represents MPA + hMG group vs control group, MPA group or hMG group. *P < 0.05.

In the MPA + hMG group, there is a significant increase in FSHR expression level compared to the control group and MPA group (P < 0.05), and  a decreased FSHR and LHR expression level compared with the hMG group (P < 0.05). Although the ER in MPA group is lower compared to MPA + hMG group, it is not significantly different between them (p = 0.0623). There is no significant differences of between all groups (P > 0.05) (Fig. 4).

### The effects of treatment on the PI3K/Akt/mTOR signaling pathway in ovarian tissue

**Figure 5:**
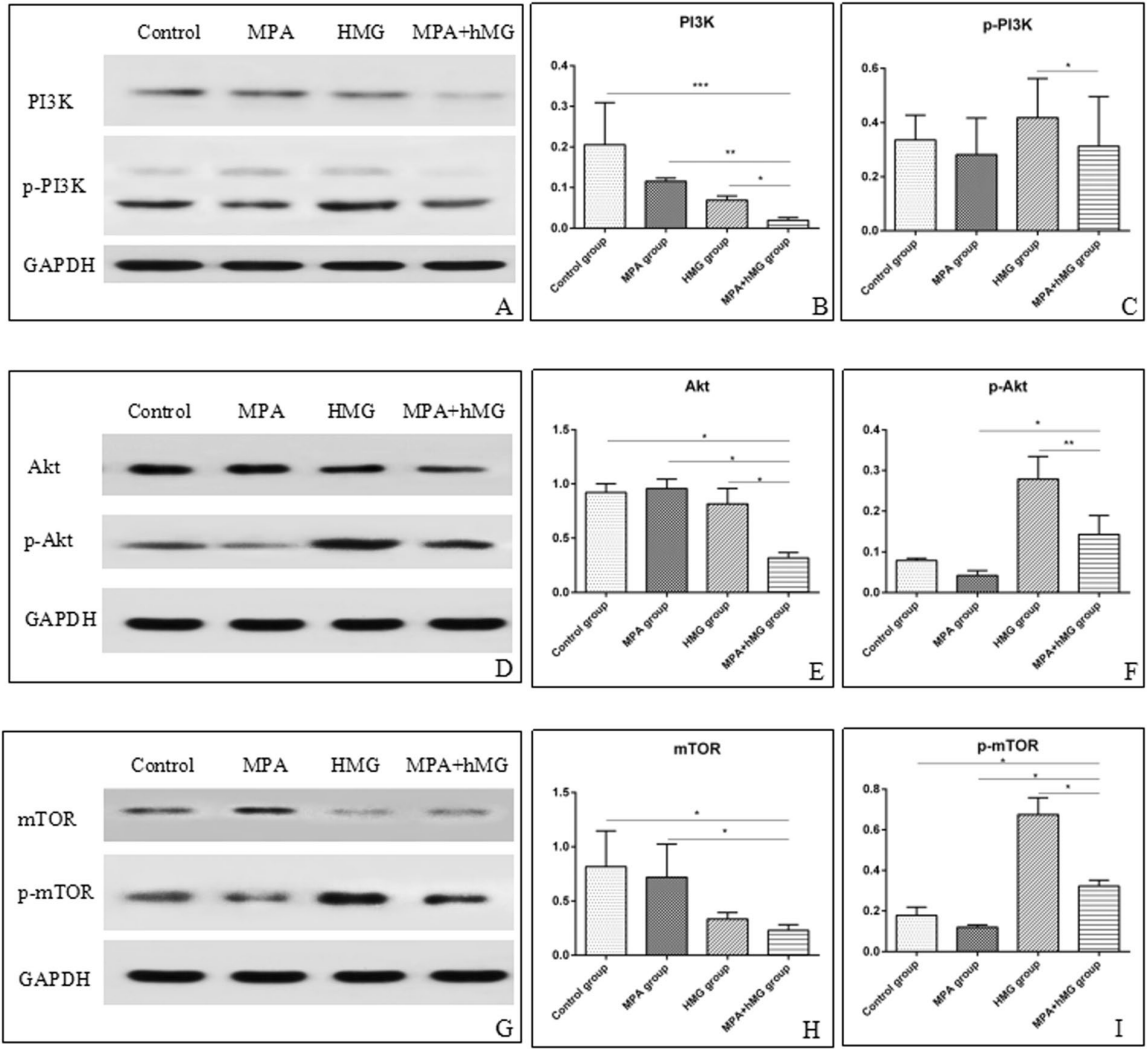
The effects of treatment on the PI3K/AKT/mTOR signaling pathway in ovarian tissue. Ten samples were used for each western blot assay, and each sample was measured in triplicate. (**A**–**C**) Western analyses of PI3K and p-PI3K, and their statistical analysis in all groups. (**D**–**F**) Western analyses of AKT and p-Akt,, and their statistical analysis in all groups. (**G**–**I**) Western analyses of mTOR and p-mTOR,, and their statistical analysis in all groups. *Represents MPA + hMG group vs control group, MPA group or hMG group. *P < 0.05.

To investigate whether the PI3K/Akt/mTOR pathway is activated by MPA + hMG treatment, we have assayed the phosphorylation of PI3K, Akt, and mTOR by western blot in ovarian tissues. Interestingly, quantification of protein expression shows that MPA + hMG causes a reduction in expression of PI3K, Akt and mTOR compared to the control group and MPA group, and the decreased PI3K and Akt in MPA + hMG group compared to the hMG group. While increased expression of p-Akt and p-mTOR in MPA + hMG group compared to the MPA group and a higher p-mTOR in MPA + hMG group than that in control group (P < 0.05). On the contrary, the expression of p-PI3K, p-Akt and p-mTOR is significantly decreased in the MPA + hMG group compared to the hMG group (P < 0.05) (Fig. 5).

## Discussion

The benefits of MPA + hMG treatment in ART to improve success rates of IVF/ICSI was recently discovered and confirmed by Dr. Kuang followed by embryo cryopreservation. However, the mechanism(s) of MPA + hMG treatment remained unclear. In this study, we generated a BALB/C mouse model of clinical MPA + hMG treatment to delineate whether this treatment have positive effects on follicular development, through regulation of ovarian hormones, their receptors and the involvement of the PI3K/Akt/mTOR pathway on follicular development during ovarian hyperstimulation. This investigation demonstrated that the serum FSH level in MPA + hMG group was higher than the control group and MPA group, and lower than the hMG group. Meanwhile, reductions of serum LH, E2 and P were observed in MPA + hMG group when compared to the control group and hMG group. Studies found that the changes of plasma FSH, E2, and P in ovarian tissues were influenced by the effect of exogenous P on the pituitary under controlled ovarian hyperstimulation^17^, and hMG can significantly increase plasma FSH and LH levels^18^. In addition, the rapid increase in E2 produces a GnRH impulse and LH surges via positive feedback of the hypothalamus and pituitary in follicular development^19^, while P has potent anti-GnRH activity exerted at the hypothalamic level. Thus, it diminishes the sensitivity of GnRH-stimulated LH release and inhibits FSH-stimulated E2 production by controlling FSH induction of the aromatase enzyme^20,21^. Thus, the MPA + hMG group still did not have a higher E2 levels during the activating PI3K/Akt/mTOR pathway. Another study found that MPA is mainly as effective as oral contraceptives to inhibit ovulation^22^, leading to the decreased of E2 and P levels in MPA + hMG group compared with the control group and hMG group. According to these results, we hypothesize that the elevated FSH and decreased LH, E2 and P were mainly attributed to the effect of hMG on FSH as well as anti-GnRH effects of the hypothalamus and pituitary mediated by MPA administration.

In the MPA + hMG group and the MPA group, the treatment of MPA resulted in remarkable ovulation inhibition and no new corpus luteum due to the use of exogenous P as a contraceptive is associated with suppression of ovulation for a longer period^23^. Interestingly, some studies suggested that the MPA treatment suppressed the dominant follicle more than the physiological dose during the growing phase^24^, there are still a number of dominant follicles in the MPA + hMG group, which may be due to the FSH, as the main component of hMG and maintenance of FSH during follicular development can result in granulosa cell proliferation and oocyte development and reduce the negative effects of MPA on dominant follicles^25^.

Analyses of ovarian hormone receptor demonstrated that a higher FSHR was showed in the MPA + hMG group than the control group and MPA group. In the current study, FSH upregulates the expression of FSHR, and the alterations in the FSHR may contribute to the variability of ovarian response to FSH^26^. In addition, high levels or activity of FSHR may affect the intensity of FSH, and in turn, the increased feedback regulation of FSHR may be stimulated by FSH^27,28^. Moreover, P increased the response of granulosa cells to FSH via increased cAMP, which plays an important role in folliculogenesis and follicle survival^29^. The fact that we observed increased FSH and FSHR in the MPA + hMG group suggested that those results might lead to the improvement of follicular growth by hMG and increased sensitivity of granulosa cells to FSH via MPA. Moreover, Karlsson *et al*. showed that the activation of LHR, upon binding LH in ovarian granulosa initiates signaling cascades that regulate transcription of genes necessary for ovulation and luteinization^30^. In our study we showed a lower LHR and non-ovulation in MPA + hMG group compared to the hMG group, which due to the P can participate in down-regulation of the LHR gene expression^31^.

Edson MA *et al*. found that FSH substantially activated the PI3K/Akt pathway in ovarian granulosa cells, and this pathway plays critical roles in folliculogenesis, including the activation of follicles and maturation^32^, and reduced fertility were found in an Akt1-knockout females, resulting in reduced numbers of antral follicles^33^. Sun *et al*. used transient treatment with mTOR and PI3K stimulators *in vitro* and observed a synergistic effect on follicular development^34^. Increasing evidence suggests the involvement of PI3K/Akt/mTOR in cell proliferation, follicular growth and development, maturation, and periodic ovulation, which is activated in oocytes and granulosa^35–37^. However, there are still limited studies that have been developed to determine whether MPA + hMG can affect follicle growth, development and maturation. In order to explore the molecular mechanisms underlying the regulatory function of MPA + hMG on follicle development, PI3K/Akt/mTOR pathway was examined in mouse ovaries. Most importantly, we found that the levels of p-Akt and p-mTOR in the MPA + hMG group were significantly higher compared to the control and MPA groups. Similar to previous studies, in this study, hMG was added to stimulate PI3K/Akt/mTOR signal transduction in granulosa cells^38^. As studies showed that FSH activated the PI3K pathway including increased Akt and mTOR, which predominantly occurs in the nucleus and cytoplasm^39,40^. This indicates that hMG can increase p-Akt and p-mTOR by activating the PI3K/Akt/mTOR signalling pathway. Our results clearly demonstrated that the development of follicles via activation of the PI3K/Akt/mTOR pathway relied on the appropriate dose of MPA combined with hMG. To our knowledge, there are no published animal trials of MPA + hMG in ovarian stimulation including an analysis of its effect on the ovary microenvironment and PI3K/Akt/mTOR signaling pathway.

Although the ovarian index was higher in the MPA + hMG group than in the other three groups, there was no statistically significant difference between the groups. This may be due to the inhibition of ovulation in MPA group, multiple follicles development but no ovulation in the hMG group, and reduced the effect of multiple follicles development and inhibition of ovulation via MPA in MPA + hMG group were presented. Therefore, the number of follicles and ovulation may affect the size of ovarian index, although in the activated PI3K/Akt/mTOR pathway.

In conclusion, our results demonstrated that follicular development in MPA + hMG treated mice were associated with activation of the PI3K/Akt/mTOR pathway and the variation of serum hormone and hormone receptor via the synergistic effect of hMG and MPA. Moreover, our findings provide a theoretical basis to establish a new protocol for ovarian stimulation for infertile patients.

## Methods

### Animal treatment

Female, 6 week-old BALB/C mice were obtained from laboratory animal center, Shanghai Jiaotong University School of Medicine and kept under standard conditions: environmental temperature 20 ± 2, relative humidity 60%~80%, and on light of 14 hours one day. Water and food were available ad libitum. Forty female mice were randomly divided into four groups including: control group, MPA group, hMG group and MPA + hMG group.

### Experimental design

The female mice cycle was observed at 8 am every day and sexual cycle was divided by the characteristic of cellular changes and selected the normal cycle mice as the experimental object. According to the principle of vaginal exfoliated cells (leukocytes, nucleated cells and keratinocytes): proestrus stage, 17–21 hours, lots of nucleated epithelium cells and a small amount of keratinocytes; estrus stag, 9–15 hours, keratinocytes; metestyus stage, 10–14 hours, leukocytes, nucleated cells and keratinocytes; ciestrus stage, 60–70 hours, only the presence of white blood cells. The control group received 0.25 ml/day salad oil via gavage; the MPA group conducted 3.0 mg/kg·d MPA (Zhejiang Xianju Co. Ltd, Zhejiang, China) via gavage; 5 IU/d hMG (Anhui Fengyuan Pharmaceutical Co. Ltd, Hefei, China) was used for hMG group via intraperitoneal injection; and the MPA + hMG group administered 3.0 mg/kg·d MPA combined with 5 IU/day hMG via gavage and intraperitoneal injection, respectively. All animals were treated for 5 days and vaginal smears were taken to determine the estrous cycle continuously.

### Ovary index calculation

Animals were sacrificed after 24 hours of MPA or/and hMG administration for 5 days. Their ovaries (bilateral) were collected carefully. The fascia and adipose tissue was removed from the ovaries and the wet weight of the ovaries were measured. Ovary index was calculated according to the following formula: ovarian index = ovarian wet weight (mg)/body weight (g) × 100%.

### Hormonal assay

Orbital blood collection were performed before treatmnet and blood collection of enucleation were performed after 5 days of treatment. Then they were kept at room temperature for 2 hours and centrifuged at 2000 g for 30 minutes. Subsequently, serum were transferred into 1.5 mL polypropylene tubes, and stored at −20 °C. Serum FSH, LH, E2 and P levels were determined using an Euzyme Linked Immimosorbent Assay Kit (Yinggong Corporation, Shanghai, China) and measured the absorbance at 450 nm.

### Histological examination

The ovaries were fixed in 4% polysorbate solution for 24 hours. Dehydration, embedding, slicing (thickness:5 um) and hematoxylin & eosin staining were performed, number of follicles at all levels were observed under an 10x magnification optical microscope. The classification of follicles refers to the classification of Myers^41^. Only follicles containing an oocyte with a visible nucleus were counted to avoid double counting.The percentage of the number of follicles at each stage on total number of oocytes was calculated.

### Tissue microarray immunohistochemistry analysis

Mouse ovaries were fixed in 10% buffered formalin for paraffin embedding and arranged by array, with an aperture of 2 mm, spacing 2 mm. FSHR (1:500, anti-mouse, Abcam, United States), LHR (1:500, anti-mouse, Boster, China), E receptor (ER) (1:400, anti-mouse, Abcam, United States) and P receptor (PR) (1:200, anti-mouse, Abcam, United States) were used to via immunohistochemistry staining. Finally, positive area analysis was performed using positive cell number expression points and according to the formula and the positive expression rate of the slice: positive rate (%) = positive number of expression/total number of cases × 100%.

### Western blotting analysis

After the mice were sacrificed, ovaries from different mice in each group were harvested and stored at −80 °C. Subsequently the protein was extracted using triple detergent lysis buffer, the expression of PI3K (CST, 1:1000), p-PI3K (CST, 1:1000), AKT (CST, 1:1000), p-Akt(CST, 1:2000), mTOR(CST, 1:1000) and p-mTOR(CST, 1:1000) was detected by western-blotting.

### Statistical Analysis

GraphPad prism 5 software was used for statistical analysis. Data was presented as the mean ± standard deviation (SD). Statistical analysis of normal distribution was performed on two independent samples. The Mann-Whitney U test was used to measure the non-normal distribution. The data were analysed by Chi-square. P < 0.05 was considered statistically significant. Assays were performed at least three times independently.

### Data availability

See ‘Availability of materials and data’ section for more information

### Ethical approval

The ethical review committee for animal experiments at the Shanghai Jiaotong University School of Medicine approved the use of mice for this study. Principles of laboratory animal care were followed and all procedures were conducted according to the guidelines established by the National Institutes of Health, and every effort was made to minimize suffering.
